# “PHE in Action”: Development and Modeling of an Intervention to Improve Patient Engagement among Older Adults

**DOI:** 10.3389/fpsyg.2016.01405

**Published:** 2016-09-16

**Authors:** Julia Menichetti, Guendalina Graffigna

**Affiliations:** Department of Psychology, Catholic University of the Sacred HeartMilan, Italy

**Keywords:** patient engagement, intervention development, older patients, chronic disease, patient activation

## Abstract

The increasing prevalence of chronic conditions among older adults constitutes a major public health problem. Thus, changes in lifestyles are required to prevent secondary conditions and sustain good care practices. While patient engagement received great attention in the last years as key strategy to solve this issue, to date no interventions exist to sustain the engagement of older chronic patients toward their health management. This study describes the design, development, and optimization of *PHEinAction*, a theoretically-driven intervention program to increase patient engagement in older chronic populations and consequently to foster healthy changes that can help reduce risks of health problems. The development process followed the UK Medical Research Council's (MRC) guidelines and involved selecting the theoretical base for the intervention, identifying the relevant evidence-based literature, and conducting exploratory research to qualitatively evaluate program's feasibility, acceptability, and comprehension. The result was a user-endorsed intervention designed to improve older patients' engagement in health management based on the theoretical framework of the Patient Health Engagement (PHE) model. The intervention program, which emerged from this process, consisted of 2 monthly face-to-face 1-h sessions delivered by a trained facilitator and one brief telephonic consultation, and aimed to facilitate a range of changes for patient engagement (e.g., motivation to change, health information seeking and use, emotional adjustment, health behaviors planning). *PHEinAction* is the first example of a theoretically-based patient engagement intervention designed for older chronic targets. The intervention program is based on psychological theory and evidence; it facilitates emotional, psychological, and behavioral processes to support patient engagement and lifestyle change and maintenance. It provides estimates of the extent to which it could help high-risk groups engage in effective health management and informs future trials.

## Introduction

Aging of the population is a major health challenge and a considerable concern for public health authorities and institutions (Ebrahim, 1997; Lutz et al., 2008). Older adults are likely to suffer from chronic diseases (Denton and Spencer, 2010), they often have multiple unmet health needs (Wolff et al., 2002; Chatterji et al., 2015), and they often have limited access to personal and contextual resources needed to purposeful engage in accomplishing health goals (Shearer et al., 2009). It is difficult for health services to meet those needs, because of the lack of resources. Consequently, renewed models of care where patients are involved as main partners of their health management are needed (Anderson and Funnell, 2005; Thomson et al., 2005). Indeed, it is becoming ever more important for patients to be partners in care, not simply recipients of care as in older paradigms, because the actions people do—as well as do not—are critical for successful disease prevention and management (Mosen et al., 2007). The existing evidences suggest that patients who are partners in care have the potential to improve health outcomes through the adoption of health-enhancing behaviors and the reduction of health inequities (Coulter, 2005, 2012; Hibbard and Cunningham, 2008; Jordan et al., 2008; Cosgrove et al., 2013; Hibbard and Greene, 2013). This is particularly true among older patients who often suffer multiple disease conditions. Moreover, engaging patients in the health management can have a pivotal role in improving the effectiveness and efficiency of care (Holman and Lorig, 2004; Remmers et al., 2009). It can also improve client's satisfaction with the care process and the maintenance of an active role in society (Mosen et al., 2007; Kubina et al., 2013). This may not only contribute to the reduction of direct costs of the healthcare system, but also concur with the (re)orientation of economic resources in the management of healthcare systems to reduce costs (Fisher et al., 2009; Remmers et al., 2009; Greene and Hibbard, 2012; Hibbard and Greene, 2013).

Not surprisingly, the importance of engaging patients in their care has been gaining increased attention from clinicians, researchers, and policymakers alike (Simmons et al., 2014; Weil, 2016). Different labels have been adopted in the scientific literature to denote the process of making patients active stakeholders of their health management (i.e., patient activation, patient empowerment, patient engagement, and patient involvement; Barello et al., 2014). Among these, the use of the term “patient engagement” has been showing an increasing trend, probably for its capacity to represent an “umbrella term” that encompasses different interconnected conceptualizations and labels (Barello et al., 2016). With this term, which is taken from the marketing literature (Hardyman et al., 2015), the dynamic relationship between the patient (“the supply”) and the healthcare system (“the demand”) and its multi-level determinants (individual, relational, contextual, organizational) are highlighted (Graffigna et al., 2014a, 2015). As showed by Graffigna et al. (2014b, p. 87), the phenomenon of patient engagement is a “multi-dimensional psychosocial process resulting from the conjoint cognitive, emotional, and behavioral enactment of individuals toward their health condition and management.” According to this definition and to other authors' explanations of patient engagement (Hibbard et al., 2004; Gruman et al., 2010; Carman et al., 2013), three main dimensions featured the patient engagement process: behavioral (the concrete actions that patients do to manage their health condition), cognitive (the thoughts and information that patients have concerning their health condition), and emotional (the feelings and emotions that patients experience when adjusting to their new health condition) (Barello et al., 2016). All these dimensions help patients become experts in managing their health and care. According to this broad conceptualization and different qualitative studies on the care experiences of chronic patients, the Patient Health Engagement (PHE) Model has been developed (Graffigna et al., 2014a,b; Barello and Graffigna, 2015; Barello et al., 2015). According to this Model, after a diagnosis of chronic illness, people move through a series of phases that express different needs for engagement in care. In a blackout phase, patients feel unable to manage their health condition and are upset. Subsequently, they can experience an arousal phase in which they perceive anxiety and worry for their condition. In an adhesion phase, they learn to manage their health condition but have problems in adjusting their health habits to new life situations. Finally, in an eudemonic phase, they feel confident in autonomously managing their health conditions, they are optimistic about their future, and they perceive themselves as the main actors of their health and their life. A 5-items unidimensional validated self-report scale (PHE-Scale) has been recently validated strongly rooted in this model, showing the ability to detect these four main patient engagement phases along the care process (Graffigna et al., 2015).

However, the findings of a recent systematic review on patient engagement interventions revealed that although the link between patient engagement and improved health outcomes has been demonstrated, few interventions exist in the literature (Simmons et al., 2014). The few existing interventions target only some components and dimensions of the patient engagement process. This could limit the evidences of such interventions. Furthermore, few studies that aimed to engage patients rarely quantified and measured patient engagement (Simmons et al., 2014). Theoretical assumptions of those studies are often weak. Additionally, little research in this area has involved older people as the main target of the research (Wetzels et al., 2007). Besides the difficulties and specific needs that older patients may have to address in self-managing their health, specific solutions might be required for this population. Despite the considerable potential of patient engagement for older adults, this field remains underdeveloped (Kane and Kane, 2001; Elliott et al., 2016). To date, little is known about how to concretely engage older patients in their health management in a way that could be integrated in the clinical practice and tailored to the older patients' specific needs and goals.

This study reports on the design, development, and optimization processes of a new theory-driven intervention program aimed at improving the engagement of older chronic patients in care management. The intervention was based on the PHE Model, because of its broader view of the patient engagement process, and it proposed the operationalization of the PHE Model in practice based on three main research phases: literature review, experts and patients' opinion.

## Methods

### Study design

The Medical Research Council (MRC) Framework was used to assist with the development and optimization of an intervention program to improve the older chronic patients' engagement in health management. This phased approach aimed to provide a robust methodological basis for the development and evaluation of complex interventions (Campbell et al., 2000, 2007; Craig et al., 2008). According to the MRC framework, prior to formal evaluation, dissemination, and monitoring of a new intervention, two main steps in the development and modeling need to be implemented to identify the theoretical base for the intervention, define contents and processes, structure the intervention, and model the procedures according to stakeholders' evaluations. In this study, this development and optimization process featured three main research phases, evidence exploration, experts' tune-up, and patients' fine-tuning. For every phase, activities, aims, and methods are described in Table 1. A small team of health psychologists responsible for the coordination and project implementation discussed the key findings of every research phase. The results of these discussions were used to set up and gradually refine the resulting intervention.

**Table 1:** **Methodological process for intervention development and modeling**.

**Phase**	**Activity**	**Aims**	**Methods**
Phase 1: “Evidences exploration”	Systematic literature review	(i) To identify existing strategies, techniques and solutions for patient engagement (ii) To match literature findings with the PHE model components	- **Scientific databases:** Medline, PsychInfo, Scopus, Cochrane. - **Search strategy:** (“patient engagement” OR “patient activation”) AND (“intervention” OR “trial” OR “program^*^”) - **Inclusion criteria**: (i) involving chronic patients, (ii) pre-/post-evaluations
Phase 2: “Tune-up with experts”	Experts' group discussion	(i) To revise, discuss and prioritize results of Phase 1 (ii) To optimize intervention's contents and procedures (iii) To collect experts' opinion about feasibility of the interventions' components	- **Participants:** 22 healthcare professionals caring for older patients trained in patient engagement theories, measures, and actions - **Procedures:** (i) presentation of literature results, (ii) group discussion to revise and optimize evidence-based results, (iii) *ad-hoc* questionnaire to evaluate feasibility, utility and adoption of an intervention for patient engagement - **Data analysis:** transcription of group discussion was thematically analyzed by members of the research team (JM, GG) to identify key issues within data (Braun and Clarke, 2006); quantitative data gathered through the evaluation sheets were synthetized with descriptive analyses by using the SPSS software 21.0.
Phase 3: “Fine tuning with patients”	Repeated qualitative semi-structured interviews	(i) To explore older patients' expectations and needs for engagement (ii) To collect patients' opinion about comprehensibility and acceptability of the intervention (iii) To collect patients' feedback to optimize the intervention	- **Participants:** 8 purposively selected patients >65 years-old affected by at least one chronic condition - **Procedures:** (i) preliminary qualitative semi-structured interview lasting about 30 minutes before the involvement in a prototype training, (ii) prototype training simulation (patients participated in a prototype version of the training), (iii) qualitative semi-structured interview lasting about 60 min just after the second session of the prototype training - **Data analysis:** Two researchers independently analyzed interviews by using audiotape transcripts of interviews. A thematic approach was adopted in order to synthetize main themes within data (Braun and Clarke, 2006).

## Results

The findings from the research phases, so as details about the resulting intervention, are presented in the following sections.

### Literature review on patient engagement techniques

The synthesized literature evidence demonstrated that interventions to engage chronic patients in their health and care management at the individual level (i.e., the patient) were generally scant, often poorly described in their components and delivery, and rarely of high methodological quality. Furthermore, they rarely targeted specifically older patients.

Despite these aspects, some key components and techniques adopted by the identified interventions to engage patients in their health and care management recurred. Thus, after selecting articles describing interventions for patient engagement, we analyzed and extracted the techniques used by the selected interventions. We thereafter summarized those techniques and synthetized them considering the three main domains of the patient engagement process:

- behavioral (the concrete actions that patients do to manage their health condition),
- cognitive (the thoughts and information that patients have concerning their health condition), and
- emotional (the feelings and emotions that patients experience when adjusting to their new health condition).

Most interventions targeted the behavioral and/or cognitive domains. Consequently, positive psychology exercises (Seligman et al., 2005; Sin and Lyubomirsky, 2009; Schueller and Parks, 2012) and expressive writing tasks (Pennebaker and Beall, 1986; Rosenberg et al., 2002; Frisina et al., 2004) were also included within the emotional part of our intervention because they showed promising results in helping patients positively adjust to their illness experience. Furthermore, the main theories and models on which the programs were based and used to elucidate the role of cognitive, emotional, and social factors in health behavior were summarized. Table 2 describes the resulting map of techniques reported in the scientific literature and used to deliver interventions for patient engagement.

**Table 2:** **Main techniques and theories emerged from literature considering the three PHE domains**.

**PHE domains**	**Techniques**	**Theories/Models**
**Behavioral**	• Goal setting and planning (Shively et al., 2005, 2013; Riegel et al., 2006; Kersten et al., 2015; Shah et al., 2015) • Motivational interviewing (Anderson et al., 1995; Riegel et al., 2006; Linden et al., 2010; Benzo et al., 2012)	Patient Activation Theory (Hibbard et al., 2004) Transtheoretical Model (Prochaska and DiClemente, 1986) Self-Regulation Theory (Leventhal, 1984)
**Cognitive**	• Question-asking tasks (Hochhalter et al., 2010; Deen et al., 2011; Maranda et al., 2014; Maclachlan et al., 2016) • Psycho-education sessions (Druss et al., 2010; Maindal et al., 2011; O'Leary et al., 2015; Krouse et al., 2016; Lara-Cabrera et al., 2016) • Salutogenesis exercises to map external resources (Tan et al., 2016) • Dai0ly diaries for self-monitoring (Nagykaldi et al., 2012; Lee et al., 2013)	Health Belief Model (Janz and Becker, 1984) Patient Activation Theory (Hibbard et al., 2004) Social Cognitive Theory (Bandura, 1986) Self-determination Theory (Deci and Ryan, 2008)
**Emotional**	• Positive psychology exercises • Expressive writing tasks • Salutogenesis exercises to strengthen inner resources (Tan et al., 2016) • Illness experience maps (Hall et al., 2015)	Stress Coping Model (Lazarus and Folkman, 1984) Salutogenesis (Antonovsky, 1996) Positive Psychology (Seligman and Csikszentmihalyi, 2014)

Personalization of actions based on baselines assessment level was also adopted in some analyzed studies to ensure that actions were tailored to participants' experiences (Hibbard et al., 2004; Rise et al., 2016).

Finally, literature reported that even relatively short interventions could increase patient engagement, and suggested to track patient engagement and specific behavioral outcomes over time using validated measures to achieve positive health outcomes (Simmons et al., 2014).

### Healthcare professionals' tune-up

Overall, 22 healthcare professionals (e.g., nurses, physicians) who were experts in patient engagement theories and strategies participated in the prototype intervention's presentation and evaluation. These were community based practitioners caring for older chronic patients in a North-Italian hospital who were previously involved in a formative program on patient engagement theories, measures, and strategies. The researchers (JM, GG) recruited willing participants, most of whom were women (72%) with the mean age of 46 years old (range: 29–62), and with the mean number of years of experience of 26 years. Evidence-based literature on patient engagement strategies/techniques and suggested exercises based on summarized techniques were presented to experts. These exercises were a prototype version of those ones described in the last section of the results and in **Table 7**. Thereafter, they were invited to discuss the presented input, to revise the proposed framework, to provide suggestions about possible ameliorations, and to identify possible advantages/barriers to the implementation of an intervention including the revised techniques. Subsequently, experts discussed a prototype of training's contents and procedures in small groups and finally evaluated the prototype intervention using a worksheet that was developed *ad-hoc* to address the following domains: easiness to use, relevance and utility, perceived competence, and willingness of adoption.

Generally, experts evaluated the proposed techniques and the framework of action positively. They particularly appreciated standardization and documentation of procedures and the possibility offered by techniques and proposed exercises to support the patient through simple and concrete steps and tasks. Thereafter, they provided suggestions and recommendations for the training's contents and procedures. The main themes that emerged from group discussion are summarized in Table 3 with corresponding quotes.

**Table 3:** **Experts' recommendations main themes and quotes**.

**Main themes**	**Quotes**
An evidence-based standardized guide to follow	*“All the proposed exercises are reported in literature and have a solid background, this helps”* *“It's easier to follow the program if exercises and procedures are well described”*
Working on multiple domains	*“I think that the most valuable aspect of the intervention could really be that it allows working on different aspects of the patient's experience”* *“I know that my patient has different need, and it's important to me to offer him actions for all his/her different needs”* *“It's true that patients need to be informed, but also to understand what is happening to them”*
Optimizing available resources	“*I usually do most of the things you described, but I do them without thinking…having a guide could help me better organizing my actions”* *“Most of the aids are already available to the patient, but he/she doesn't know that there are and how to use them”*
Supporting good communication exchanges	“*Some exercises are like a guide for our exchanges with the patient”* *“This can help having a guide for my communication with the patient…I already ask to my patient his story, but with some of these exercises I can have some practical tool”*
A tool to create bridges	“*Results of assessment and exercises can be used by other colleagues to continue the work with the patient”* *“Patients can feel accompanied by professionals also outside the hospital bridges, it can be a way to stay in touch with the patient”*
Motivating patients to change	“*the process of patient engagement could require a pre-existing degree of motivation on the individuals' part”* *“it's important to consider motivation of patients”*
Autonomy vs. presence	“*the illness experience map is useful if you use it in the first encounter with the patient”* *“I think that this intervention is easily suitable and implementable in clinical practice, but time and spaces are surely a potential barrier…I appreciate that some exercises can be autonomously managed by the patient”* *“Is useful to give to the patient some at-home exercises, it could be a way for the patient to bring patient engagement into the home walls”*
Supporting a patient-centered organizational culture	“*we are speaking about a cultural change”* *“we need an organizational structure supporting the introduction of a similar training”* *“looking only to the disease can be a barrier, professionals should be trained to support a patient-centered culture”*
Working in tandem with patients and caregivers	“*you know…most of my patients are older…some of them have a low educational level, live far from the hospital, or have impairing conditions…what about a training also for caregivers?”* *“I think it could be useful for our population of patients to train also caregivers”*

**Figure 1:**
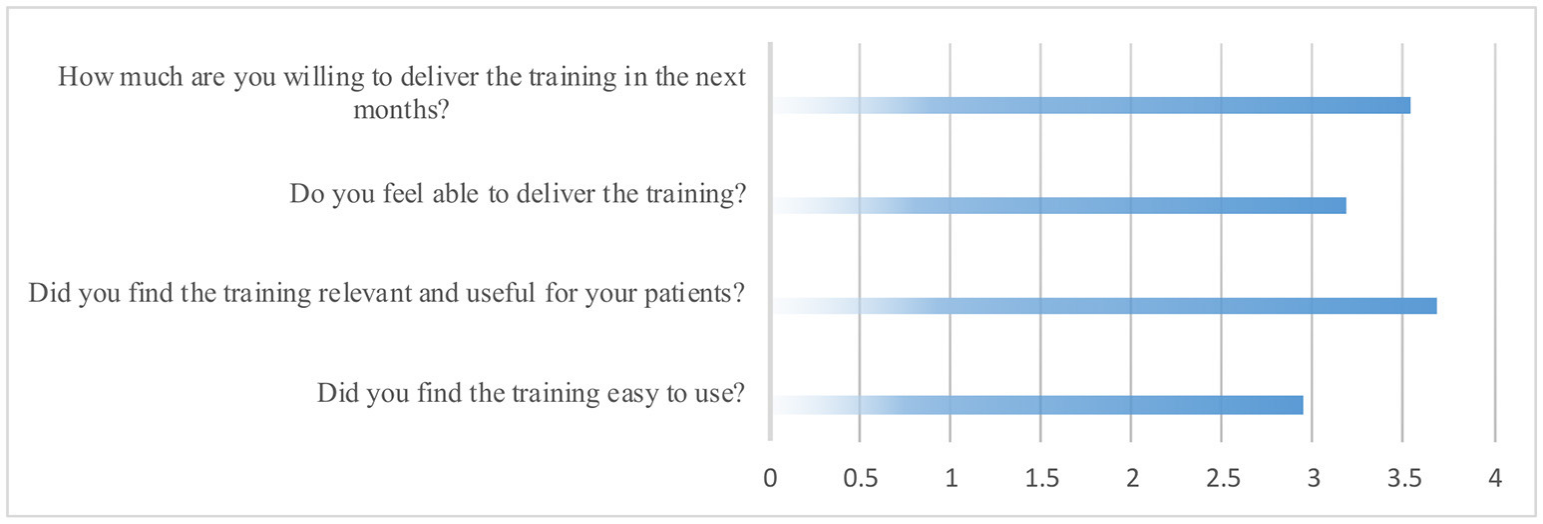
**Healthcare professionals' evaluations responses**.

Finally, on average, experts rated the proposed prototype intervention on a 5-point Likert scale as highly relevant and useful (ranging from moderately to extremely), and they reported high willingness to implement the training in their clinical practice (ranging from moderately to extremely). They generally evaluated the exercises as moderately easy to use (ranging from slightly to very) and felt moderately able to deliver them (ranging from slightly to very) (see Figure 1).

#### Listening older patients: fine tuning the intervention

Eight participants older than 65 years accepted to participate in the “fine tuning” phase. Those patients were purposively and sequentially recruited by an external researcher through community senior centers, senior associations, or community medical centers. No exclusion criteria with the exception of age (>65 years old) or diagnosis (>1 chronic health condition) were applied. Most participants were women (72%), affected primarily by type 2 diabetes (57%). Most had an elementary education (57%), and indicated that they were married (71%). All of them were retired. Three participants were overweight, but none of them was a smoker. Almost all participants had multiple diseases and comorbidities, mainly with cardiovascular disorders. For further details about the characteristics of the sample, see Table 4.

**Table 4:** **Characteristics of participants (*n* = 8)**.

	**mean (*SD*)/ *n* (%)**
Age	73 (4)
**SEX**	
Male	2 (25%)
Female	6 (75%)
Education (years)	8 (4)
**DIAGNOSIS**	
Type 2 diabetes	4 (50%)
Cardiovascular disease	2 (25%)
Chronic respiratory disease	1 (12,5%)
Inflammatory bowel disease	1 (12,5%)

Participants completed two face-to-face semi-structured qualitative interviews conducted in their homes just before and just after the involvement in a simulation of the prototype training (this was a second generation prototype version of the training modified based on the experts' revision of the primary generation prototype training) and participated in the presentation of the training's materials and procedures (see Table 5 for further details about the interviews' track).

**Table 5:** **Interviews' guide**.

**Area**	**Exemplificative questions**
**PRELIMINARY INTERVIEW**	
Experiences and needs for engagement	• In your experience, what would help one in being more engaged in managing his/her health? What would help you in being more engaged? • Would you be interested in participating in initiatives aimed to foster your engagement in health management? Why would you engage in efforts to be more involved in your health management?
Expectations for a patient engagement intervention	• What features should have an intervention to engage you in managing your health? • Which contents and which way of delivery would you prefer?
**SECONDARY INTERVIEW**	
Patients' experiences about their participation in the intervention	• Could you describe me the main reasons which bring you in participating in the intervention? • Could you describe what happens in the sessions, in your own words? • How did you feel when you participate in the intervention? How did you feel before and after starting the sessions? • If you had to describe what the intervention means to you, what would you say? What images/metaphors come to your mind?
The intervention effects on the daily life and on health management	• In your opinion, how the intervention improved your engagement and attitude toward managing your health? How did it affect your daily life? • If you think about your way of managing your health, what aspects of the intervention have contributed to it? How? • If you think about your daily life and lifestyle, what aspects of the intervention have contributed to them? How?
Intervention satisfaction and feedbacks	• How would you rate and define your satisfaction toward the intervention? What aspects satisfied you more? What satisfied you less? Why? • What were the obstacles and difficulties? • What would you change or improve?

The first interviews round aimed to elicit the needs and expectations for engagement among participants. All participants reported interest in participating in an intervention designed to engage them in their health management with different motivations (“*to become more calm, I'm too anxious when I manage my health,”* “*to succeed in changing my lifestyle according to my health condition,”* and “*I'm really upset, I need to understand what it is happening to me and reorganize my life”*). They did not have particular expectations for a patient engagement intervention. Most of them (57%) preferred an individual intervention.

After this preliminary interview, patients were invited to participate in a simulation of the prototype training and to adopt the developed at-home exercises in their daily life. Patients were also asked to complete a battery of questionnaires before and after the prototype training to evaluate the potential compilation burden and the feasibility of measures. Participants were informed that the training would have been refining basing on their feedbacks and invited to point out possible difficulties with comprehension or other aspects of the program. One month after the preliminary interview and the involvement in the first prototype session, patients participated in the second prototype intervention session followed by a second round of interviews in which materials and procedures were discussed with patients.

The responses to the intervention were generally positive. Adherence to the home practice was high, as all participants used all of the provided instruments and engaged in a sustained effort to pursue their health goals. Table 6 summarizes the main themes that emerged from this second round of interviews.

**Table 6:** **Main themes and quotes of patients' perception of the intervention**.

**Main themes**	**Quotes**
A new perspective to the disease	*“I already knew all these things, but I kept them insight myself and gave no importance to them, with this intervention I eviscerate them and thus I faced them”* (Int. 1, F, 77 years-old) *“It was important to me because I slowed down and I reflected on my situation, it was difficult but important and satisfactory”* (Int. 6, F, 68 years-old) *“it is now for me like some light into the fog is appearing…and this light changes your perspective…it is like I'm realizing some things”* (Int. 2, F, 69 years-old)
A stimulus to change	*“it was useful for me to manage my emotions and my anger…it helped me”* (Int. 5, F, 70 years-old) *“I want to thank you because I never thought to be able to do something to better manage my health”* (Int. 2, F, 69 years-old) *“it was like a flowered field with the sun…this pathway made me serene”* (Int. 1, F, 77 years-old)
Clinicians have to play their part	“*doctors did not play their part”* (Int. 3, M, 76 years-old) “*asking questions to my doctor is hard, I prepared myself for the doctor's visit but the encounter was brief and it ended on the hoof”* (Int. 2, F, 69 years-old)
Improving repetitiveness and comprehensibility	*“sometimes home-works appeared repetitive and I would have benefitted by more concise exercises”* (Int. 6, F, 68 years-old) *“I only completed the elementary degree so some words were difficult to me to understand”* (Int. 5, F, 70 years-old)

The average response for recommending the training to another person was high. In particular, participants indicated that they would suggest the intervention to just diagnosed people and to people who are less interested in managing their health, although those people were also perceived as difficult to engage in the intervention.

### “PHEinAction”: an intervention for older people health engagement

According to the main results of the previously described research phases, the prototype version of the training, which was outlined based on the PHE theory and literature analysis, was finally refined.

The resulting intervention was an individual training consisting of 2 monthly 1-h sessions, one brief telephonic consultation between the two sessions, and a set of instruments to be used by participants at home. Each encounter contributed to the promotion of manageability and meaningfulness disposition to manage health.

**Figure 2:**
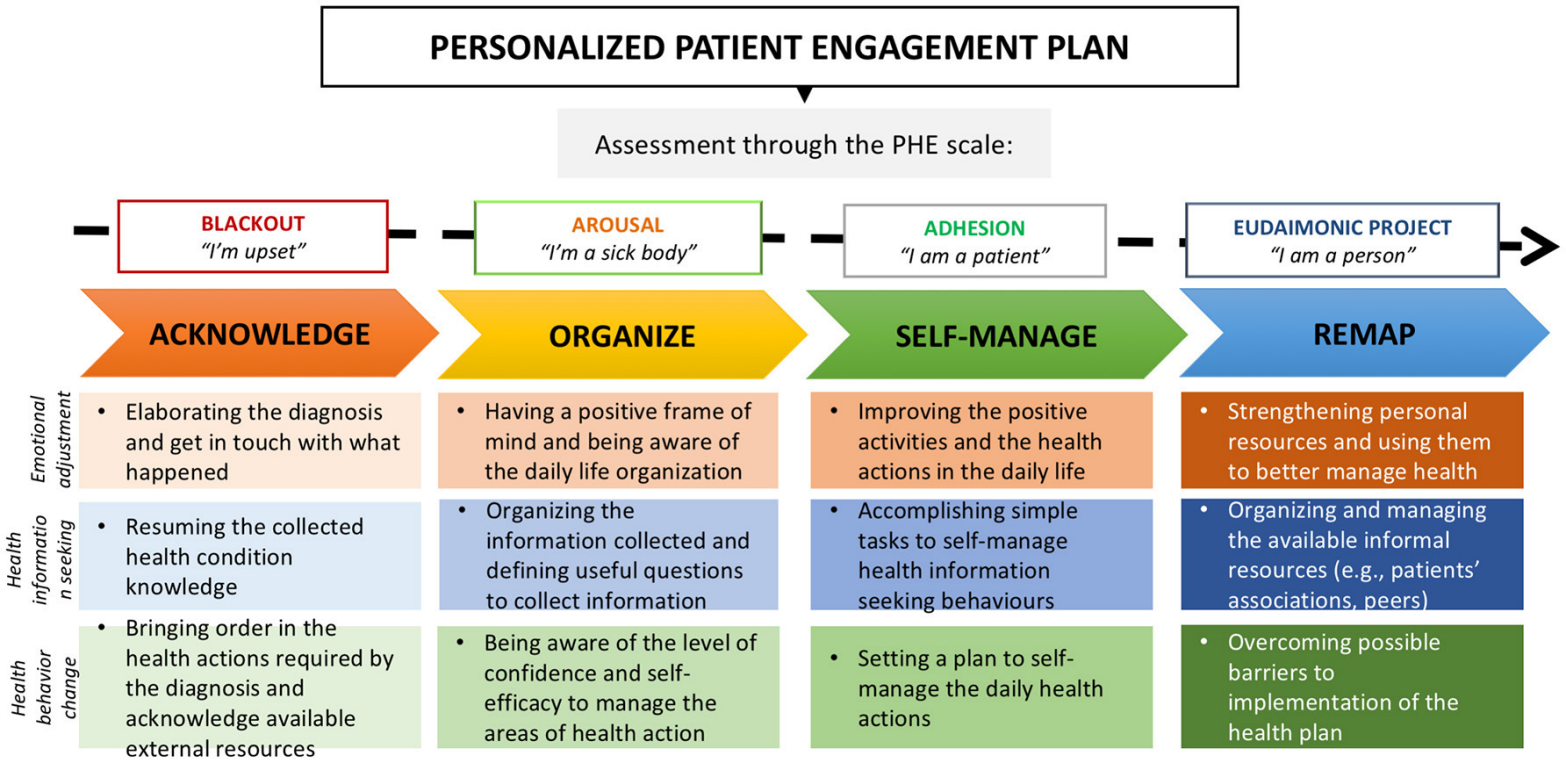
**The personalized patient engagement plan: Goals of the four training's paths basing on the baseline PHE phase**.

The first face-to-face session was used to (i) collect information on the patient's background through a patient's experience map, (ii) assess the starting phase of engagement of participants (i.e., blackout phase, arousal phase, adhesion phase, eudaimonic project phase) through a structured and validated questionnaire (The PHE Scale of Graffigna et al., 2015), (iii) define a purpose of engagement and manageable behaviors to sustain this purpose, and (iv) administer the instruments to support those purpose and behaviors based on the baseline PHE phase. Thus, for every phase of engagement, an individually tailored goal with subsequent emotional, informational, and behavioral actions was defined. Goals and behaviors defined in the individual sessions were based on the PHE phase of participants and were driven by PHE theory. The contract regarding the defined goals and behaviors was made with participants according to their particular needs and expectations for care. Furthermore, for every goal and actions, a set of instruments was developed to sustain the PHE process at home. Those instruments were developed based on the literature review of the existing strategies/techniques adopted to engage patients in their care management (see Table 2) and on experts and patients' feedbacks. Following the PHE theory, they covered three main areas of action (i.e., emotional adjustment, health information seeking and use, and health behavior change). For every area, instruments were personalized to each of the four PHE phases, yielding four packages of instruments that became increasingly challenging across phases. Indeed, as suggested by the literature and endorsed by experts, personalization of actions based on PHE phases and consequently on patients' needs and desires for care was considered a key aspect of the intervention. Four different paths of training, with specific goals and consequent specific selection of exercises related to each area of action, were thus featured to enhance flexibility and personalization of the intervention (see Figure 2 for further details about the main goals of the four training's paths based on the baseline PHE phase of participants). During the first session, a personalized PHE plan was thus defined with home-based exercises. Participants were invited to follow their plan in the next month and to actively adopt instruments of their plan to reach their engagement goal. Table 7 provides further details about the instruments' aims and contents.

**Table 7:** **“PHEinAction” home-based instruments' key components, aims and procedures**.

**Instruments**	**Aims**	**Exercises**
Instrument 1. Emotional adjustment	• To foster a process of adjustment to the diagnosis and to the patient's role • To activate and strengthen skills and inner resources of the patient	1) Expressive writing exercise on the illness experience; 2) Daily diary with small positive thinking tasks; 3) Map of wellbeing/discomfort areas in the daily life and strengthening exercise of wellbeing areas; 4) Positive psychology exercise to identify 3 personal strengths and apply them to better manage health
Instrument 2. Health information seeking and use	• To improve health information seeking/use processes • To sustain the adoption of external resources	1) Disease knowledge elicitation exercise; 2) Question-asking exercise; 3) Prompts to self-manage information-seeking behaviors; 4) Map of adopted informal information channels (e.g., internet, peers, books…)
Instrument 3. Health behavior change	• To sustain the plan and organization of health behaviors • To improve self-efficacy in managing health	1) Map of areas of action that patient needs to manage (diet, physical activity, medications…) and of informal resources supporting the management of these areas; 2) Self-evaluation exercise concerning self-efficacy level for every area of action that patients need to manage and identification of reasons for self-evaluation; 3) Behavioral plan to activate health actions; 4) Imagination exercise of possible barriers getting in the way for the plan and of solutions to handle these barriers

A telephonic consultation was conducted 2 weeks after the first encounter to maintain motivation of participants and discuss potential difficulties.

Finally, a second face-to-face session was used to (i) collect the experience of participants and discuss the adopted instruments, (ii) re-assess the PHE phase of participants through the PHE-Scale, (iii) provide feedbacks and reinforce improvements, and (iv) define a new engagement goal with related actions and instruments.

**Figure 3:**
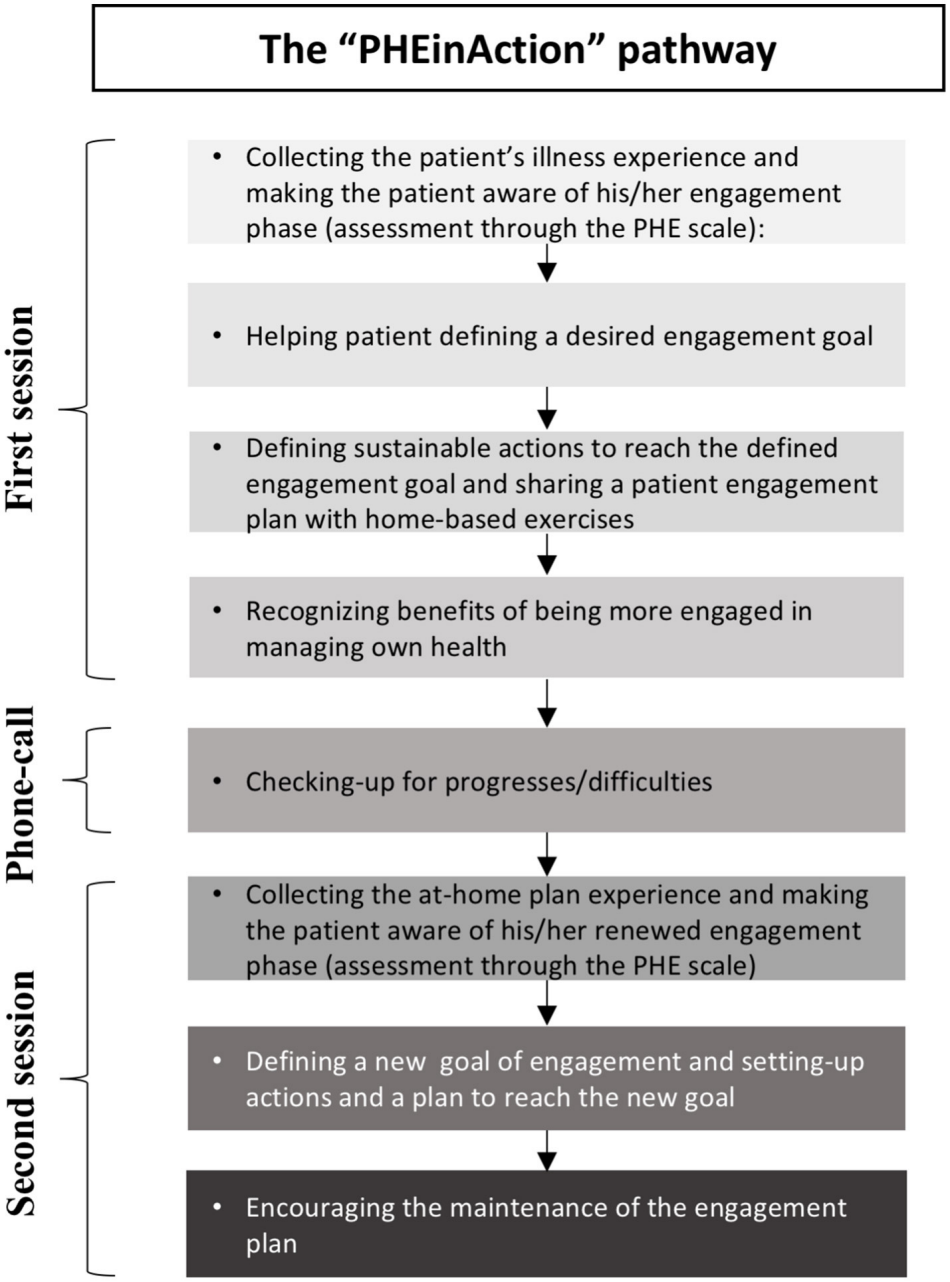
**The “PHEinAction” pathway: training structure and main sessions goals**.

Figure 3 provides further details about the structure and the sessions' goals of the final intervention.

These two sessions were conceived to be the minimal unit of action for the patient engagement change, and further “units of actions” were suggested in critical points of the care process to sustain the change process.

## Discussion

This paper describes the process of the development and refinement of a theory-based individual training aimed to engage older chronically ill patients in their health and care management. To our knowledge, this is the first intervention that aims to sustain health engagement among older chronic population. The development process allowed to define, refine, and optimize the contents of the training. Overall, patients and healthcare professionals provided positive feedbacks for the training contents and procedures. The training still requires formal evaluation.

The development process highlighted some key points that to our opinion need to be discussed.

First, although the training was initially developed for patients affected by different chronic conditions, and no differences in the fruition of the intervention among the different clinical conditions were found, it might need to be adjusted to specific chronic conditions to enhance potential benefits of the training. Even more, the results revealed that the few months following the diagnosis can represent an optimal window of action to deliver the training. This is supported by the literature suggesting that the period just after diagnosis is particularly important to allow a process of diagnosis adjustment to be started (de Ridder et al., 2008). This should be tested by further studies and evaluations.

Second, experts envisaged families and caregivers as crucial to sustain patient engagement, especially in situations in which patients are physically compromised. Probably, a training could be specifically developed to engage also caregivers and families in the care process to better sustain patient engagement. As highlighted in the literature, giving support, and engaging families and caregivers could help ensure high-quality care at home (Wellard and Street, 1999; Donelan et al., 2002), thus strengthening the engagement of patients and supporting them when directly engaging them is hindered by physical or contextual barriers.

Third, clinicians were described by patients as a potential barrier to change in patient engagement. Complex programs that train clinicians to embrace requests of patients for engagement and, even more, enable patients to become partners in their care management could make patient engagement more effective. The role of clinicians in advancing the patient engagement have been increasingly emphasized, as they play a crucial role in guiding patients on their care journey (Greene and Yedidia, 2005; Killaspy et al., 2015).

Finally, it is important to consider that this study aimed to report the development and refinement process of a new intervention, rather than to quantitatively evaluate its effect on validated measures. More data are needed to demonstrate the effectiveness of the intervention, especially in the long term, and to drive conclusions. The limited sample size (and the broadness of the inclusion criteria) did not allow us to make conclusions on the efficacy of the program. Patients' views expressed in this study were from a particular sample, mainly woman, and it was difficult to retrieve recently diagnosed participants. Furthermore, patients' feedback recorded in this study was qualitative in nature, and as such, it needs to be complemented by quantitative evaluations. The intervention needs to be further evaluated using a larger sample of males and females with different degree of engagement at baseline and including also recently diagnosed patients. It would be advisable to test the feasibility of the intervention in more homogeneous populations. Furthermore,—although this was not the primarily aim of this study—preliminary results collected through the PHE Scale suggested few changes in the engagement scores of patients enrolled in the intervention. In particular, only patients with lower levels of engagement at baseline improved their scores after the training. Participants reporting high levels of engagement at baseline (equal to or greater than the adhesion phase measured through the PHE scale) generally maintained their baseline scores after the training. This is consistent with other studies showing that particularly less engaged patients might benefit from activation interventions (Deen et al., 2011). A more systematic effectiveness study is needed to explore the stability of these first preliminary results. Additionally, changes in engagement scores might need more time to be detected, and follow-up evaluations could be particularly relevant when conducting a similar training.

To conclude, this study described the development process and optimization of a new individual intervention program to engage older chronic patients in their care management. The study utilized a step-wise structured approach to develop complex interventions (MRC) and a theoretical model based on qualitative studies and grounded on the specific needs of the target group. Indeed, grounding health interventions on qualitatively-based theories and adjusting them to the specific needs and context of final users can help deliver ecological studies. The intervention components, developed and evaluated by experts and older patients, were considered feasible and acceptable as well asuseful and easily implementable in clinical practice. Some suggestions for changes in health actions and attitudes after the training were also envisaged. Further work is needed to improve and adapt the intervention components and tackle issues related to their delivery and implementation within healthcare professionals' existing clinical practice.

## Author contributions

GG and JM designed the study and developed the methodology. JM collected the data, performed the analysis, and wrote the manuscript. GG supervised the data collection and analysis process, and revised the manuscript.

### Conflict of interest statement

The authors declare that the research was conducted in the absence of any commercial or financial relationships that could be construed as a potential conflict of interest.
